# Classification of Cochrane Plain Language Summaries by Conclusiveness Using Transformer-Based Models and ChatGPT: Retrospective Observational Study

**DOI:** 10.2196/72657

**Published:** 2026-04-14

**Authors:** Antonija Mijatović, Luka Ursić, Nensi Bralić, Ružica Bandić, Barbara Ćaćić, Ivan Buljan, Ana Marušić

**Affiliations:** 1 Department of Research in Biomedicine and Health, Centre for Evidence-based Medicine, University of Split School of Medicine, Šoltanska 2A, Split, 21000, Croatia, 385 21557820; 2 Faculty of Humanities and Social Sciences, University of Split, Split, Croatia; 3 Department of Psychology, Faculty of Humanities and Social Sciences, University of Split, Split, Croatia

**Keywords:** plain language summary, PLS, large language models, Scientific Bidirectional Encoder Representations from Transformers, SciBERT, Longformer, fine-tuning

## Abstract

**Background:**

Cochrane plain language summaries (PLSs) aim to make systematic review findings more accessible to the general public. However, inconsistencies in how conclusions are presented may impact comprehension and decision-making. Classifying PLSs based on conclusiveness can improve clarity and facilitate informed health decisions.

**Objective:**

This study aimed to develop and evaluate deep learning language models for the classification of PLSs according to 3 levels of conclusiveness (conclusive, inconclusive, and unclear) and to compare their performance with a general-purpose large language model (GPT-4o).

**Methods:**

We used a publicly available dataset containing 4405 Cochrane PLSs of systematic reviews published until 2019, already classified by humans according to 9 categories of conclusiveness regarding the intervention’s effectiveness or safety. We merged these categories into 3 classes based on the strength of conclusiveness: conclusive, inconclusive, and unclear. For the fine-tuning, we used Scientific Bidirectional Encoder Representations from Transformers (SciBERT), a pretrained language model trained on 1.14 million papers primarily from the health sciences, and Longformer, a transformer model designed specifically to process long documents. The script was developed using the Python programming language and the PyTorch framework. We computed evaluation metrics using the *scikit-learn* machine learning library and determined the area under the curve of the receiver operating characteristic (AUCROC) to measure the model performance in balancing sensitivity and specificity. We also analyzed a separate set of 213 PLSs and compared the predictions of our pretrained models with both manual verification and outputs generated by ChatGPT.

**Results:**

The model based on SciBERT achieved a balanced accuracy of 56.6%. The AUCROC was 0.91 for “conclusive,” 0.67 for “inconclusive,” and 0.75 for “unclear” conclusiveness classes. The Longformer-based model had a balanced accuracy of 60.9%, with AUCROCs of 0.86 for “conclusive,” 0.67 for “inconclusive,” and 0.72 for “unclear” conclusiveness classes. Both models underperformed compared with ChatGPT, which demonstrated higher accuracy (74.2%), better precision and recall, and a higher Cohen κ (0.57).

**Conclusions:**

Fine-tuning 2 transformer-based language models showed mixed results in classifying Cochrane PLSs by conclusiveness, likely due to semantic overlap and subtle linguistic differences. Despite satisfactory internal test metrics, the fine-tuned models failed to generalize to newly published PLSs, where performance dropped to near-chance levels. These findings suggest that general-purpose large language models like GPT-4o may currently offer more reliable results for practical classification tasks in biomedical applications.

## Introduction

A Cochrane plain language summary (PLS) is a stand-alone summary of a Cochrane systematic review used to disseminate health-related evidence to a wider audience with the goal of facilitating evidence-based decision-making about health care, particularly for medical treatments [1]. A well-written PLS should be comprehensible to readers without a background in research or health care, including patients, caregivers, and policymakers [2], and should be presented at or below a sixth-grade reading level to ensure accessibility for all readers [3]. It should also allow readers to comprehend the certainty of evidence and to correctly interpret the results, which is why the authors should not offer specific recommendations but rather present their findings clearly and guide the readers toward independent conclusions [4,5]. However, a PLS necessarily has a conclusion section conveying the main message, where the level of certainty of the evidence is presented using narrative statements [2]. For example, “Intervention causes a large reduction/increase in outcome” is a suggested narrative for large effect size and high certainty of the evidence, whereas “It is unclear if intervention has an effect on outcome” should indicate very low certainty [6,7].

Conclusiveness is an important concept in research and health care, indicating a degree of confidence in the findings and facilitating decision-making [8,9], while also ensuring that the current evidence is easily understood [10]. When provided with conclusive health information, patients rely less on health care professionals to decide on diagnosis and therapy [11,12]. Unfortunately, they do not always succeed in finding relevant information for their health condition, as shown in a study where a quarter of respondents did not find answers to the health-related inquiries they raised on the internet [13]. Patients also often find information in unreliable sources, providing misleading or false data on diagnostics and treatment [14]. In addition, numerous studies have reported higher levels of anxiety and cyberchondria with increases in online health information searches [15-17]. Given that Cochrane Reviews represent the best available knowledge in the field [1], the accurate classification of their PLSs could significantly improve patients’ comprehension of these conclusions and allow them to make well-informed decisions about health care interventions.

Several studies and reviews on the conclusiveness of Cochrane PLSs found that 50% to 80% of the reviews enabled readers to reach a relevant conclusion, while the readability of PLSs was generally poor, with conclusions often unclear or missing [18-22]. However, in some cases, conclusive statements were made even when the quality of evidence was low or moderate [23]. Our previous research showed that most PLSs lacked or had unclear conclusions regarding an intervention’s efficacy and safety [24].

In all of the studies on the conclusiveness of systematic reviews, the process of classifying the reviews and PLSs was carried out manually, usually by at least 2 independent assessors, which is a demanding and time-consuming task. In this study, we explored whether the classification of PLSs according to their level of conclusiveness could be conducted with the help of artificial intelligence (AI) and natural language processing (NLP). NLP algorithms, particularly deep learning models like neural networks, can automatically learn and extract patterns from language data [25], enabling them to understand context and semantics [26,27]. Their multilingual variants continue to expand and become accessible to speakers of less-represented languages [28] while domain-specific models lead to greater accuracy [29].

## Methods

### Overview

In this retrospective observational study with a supervised machine learning approach, we used a pretrained deep large language model (LLM) for PLS classification according to 3 levels of conclusiveness: conclusive, inconclusive, and unclear. We fine-tuned 2 transformer-based models—Scientific Bidirectional Encoder Representations from Transformers (SciBERT) and Longformer—for our task. SciBERT is a variant of the Bidirectional Encoder Representations from Transformers (BERT) model specifically designed for scientific and biomedical text processing that is pretrained on a vast corpus of scientific literature consisting of 18% of papers from computer science and 82% from the biomedical field [30]. Longformer is a transformer architecture optimized for processing long documents through sparse attention mechanisms [31]. SciBERT was selected to leverage the domain-specific language of PLSs, whereas Longformer was selected to accommodate PLSs with extended length. Specifically, the median number of words in a PLS is 345 [32], which corresponds to approximately 500 tokens, just under SciBERT’s 512-token limit. However, the Longformer model’s extended token capacity of up to 4096 tokens allows for the processing of all PLSs without truncation.

### Data Source

We used the dataset from our previous study [24], which contains 4405 Cochrane PLSs of systematic reviews on intervention studies published until 2019, already classified by 2 independent experts into 9 categories based on the conclusiveness regarding an intervention’s effectiveness or safety. We combined these categories into 3 distinct classes: conclusive, inconclusive, and unclear, allowing for a more manageable and interpretable classification task (Textbox 1).

Textbox 1. **Classification of conclusiveness categories.**

**Conclusive**

- Positive: signifies the existence of moderate- or high-quality evidence supporting the effectiveness or safety
- Negative: indicates the presence of moderate- or high-quality evidence of intervention’s ineffectiveness or harm
- Equal: denotes that the analyzed interventions were of equal effectiveness or safety

**Inconclusive**

- Positive inconclusive: implies the existence of evidence supporting effectiveness or safety, yet the evidence is low quality or inconclusive. The authors suggest that more research is needed.
- Negative inconclusive: suggests there is evidence of ineffectiveness or harm (indicating that the observed effect or the intervention was unsafe), yet the evidence is low quality or inconclusive. Authors may advise against the intervention or comparison and state that more research is required.
- Equal inconclusive: indicates that the interventions exhibit comparable levels of effectiveness or safety, yet the evidence is low quality or inconclusive. The authors emphasize that more research is required.

**Unclear**

- No opinion: the authors provided no opinion.
- No evidence: there is no evidence from randomized controlled trials because the literature search did not result in any eligible studies (ie, empty reviews).
- Unclear: the authors did not present clear conclusions.

With the classification, the evidence in the “conclusive” class is strong and clear, irrespective of the direction of the effect, as opposed to the “inconclusive” class, where it is uncertain or of lower quality. In the “unclear” class, conclusions are absent, either because authors have not provided a clear opinion or due to a lack of available evidence. This lack of conclusiveness is not indicative of a poorly written PLS, as long as the PLS accurately represents the findings from the systematic review. This is why we must differentiate between PLSs that conclude that there is “no evidence” and those that offer no opinion or present unclear conclusions [24].

### Language Processing Models

LLMs are important components of NLP designed to understand and generate human language. LLMs such as GPT-3 and BERT are pretrained on massive datasets containing text from the internet [33,34]. Most importantly, they are highly adaptable, meaning they can be fine-tuned for specific tasks [35]. For example, Beltagy et al [30] fine-tuned BERT, an LLM that had been pretrained on a wide range of text on the internet [33], to develop SciBERT, an LLM trained on a vast corpus of scientific literature, primarily from biomedical and life sciences, making it suitable for NLP tasks in the scientific and medical research domains. Similarly, Longformer was developed to address the limitations of handling long documents; it uses a sparse attention mechanism, where each token focuses on a limited local context rather than the entire sentence [31].

We achieved transfer learning by further fine-tuning SciBERT and Longformer on our specific PLS classification task. In transfer learning, the LLM adapts its learned features to the nuances of the new task while retaining the knowledge it acquired during pretraining [36]. This approach is intended to mirror how humans learn, as we often apply knowledge and skills acquired in one context to solve new, related problems [37]. Transfer learning not only speeds up the training process but also leads to better performance compared with training from scratch [38].

### Experimental Setup and Fine-Tuning

We wrote the script using the Python programming language (version 3.12.3; Python Software Foundation) with the help of the PyTorch framework [39] and executed it within the Jupyter Notebook environment [40] using the NVIDIA GeForce RTX 3080 GPU (version 8200).

Both of our models came from the Hugging Face library [41]. For SciBERT, we used its associated tokenizer, setting the maximum token length to 512. For Longformer, we extended the maximum token length to 2048 to accommodate the full content of the PLSs without truncation. Both models included a dropout layer with a rate of 0.5, a regularization technique that reduces the risk of overfitting.

For both models, we used a 768-dimensional pooled embedding vector as input to our classifier. This representation was passed through a dropout layer and a linear layer that produced a 3D output corresponding to our target classes. SciBERT used its built-in pooled ([CLS] token) representation, whereas Longformer used mean pooling across all token embeddings due to the absence of a pooler layer. A standard attention mask was applied during encoding, ensuring that padding tokens were fully excluded from self-attention computations. We used AdamW as the optimizer to update network weights [42] and categorical cross entropy as our loss function [43]. We set the maximum number of training epochs (where 1 epoch represents a complete pass through the training dataset) to 15 for SciBERT and 10 for Longformer, with early stopping based on validation loss. In practice, SciBERT training stopped after 7 epochs due to early stopping. The learning rate was set to 2×10⁻^5^ for both the models. Since these hyperparameters cannot be determined a priori, they were selected via trial and error [44]. For this reason, we monitored training and validation performance and used early stopping based on validation loss to prevent overfitting. The best-performing models (lowest validation loss) were saved and later used for evaluation.

Baseline models (SciBERT and Longformer) were implemented using a frozen feature-extraction transfer-learning approach. All pretrained transformer encoder weights were frozen, and only the newly added linear classification layer was trainable. The encoder itself did not undergo any gradient updates. Therefore, this baseline represents a lightweight transfer-learning model.

### Data Splitting and Handling of Class Imbalance

We divided the dataset into training (80%), testing (10%), and validation (10%) subsets. Random undersampling was applied only to the training set, where the smallest class contained 343 PLSs. All classes were downsampled to this size to create a balanced training set.

### Model Validation

To assess performance, we used functions from *scikit-learn* [45], including *balanced_accuracy_score*, which calculates balanced accuracy for addressing imbalances in multiclass datasets, and *precision_recall_fscore_support*, which provides precision, recall, and F-beta scores for each class. Precision is the proportion of true positives relative to the total of true positives and false positives. Recall is the proportion of true positives relative to the total of true positives and false negatives. The *F*_1_-score is the harmonic mean of the precision and recall. Additionally, we evaluated the model’s ability to balance sensitivity and specificity by measuring the area under the curve of the receiver operating characteristic (AUCROC), which is the proportion of area below the receiver operating characteristic curve, which in turn is the plot of the true positive rate against the false positive rate.

### Effect of Training and Validation Split on Model Performance

To evaluate the impact of different training and validation splits on model performance, we evaluated SciBERT’s performance with 10%, 20%, and 30% of the data reserved for validation. This analysis was conducted to assess the relationship between training set size and classification accuracy, based on the assumption that larger training sets may improve model learning. By progressively reducing the number of training samples, we tested the extent to which performance would degrade with less training data.

### Manual Validation and Comparison With GPT-4o Performance

To evaluate model performance, we used a separate verification dataset consisting of 213 Cochrane PLSs published between September 2024 and May 2025, which was not a part of the original training or evaluation datasets. Each PLS was independently classified by 2 domain experts, with a third expert resolving any discrepancies (NB, a metascientist with expertise in Cochrane PLSs and conclusiveness assessment; RB, a research assistant at the Department of Research in Biomedicine and Health; and BĆ, a psychology student with experience using AI tools to assess compliance with reporting guidelines).

The GPT-4o model was prompted using a zero-shot classification approach, which means no example classifications were provided in the prompt. The prompt instructed the model to classify each PLS into one of 3 predefined categories and included detailed definitions for each class, identical to those used by human annotators. The complete prompt can be found in Multimedia Appendix 1.

The performance of the 2 trained BERT-based models—SciBERT and Longformer—was compared with the baseline GPT-4o model (subscription-based) and evaluated against labels assigned by human experts. Model outputs were compared using standard classification metrics: accuracy, precision, recall, *F*_1_-score, and Cohen κ, with the lattermost being used to assess the level of agreement between model predictions and the expert consensus categorization.

### Calibration Analysis

To evaluate the reliability of predicted probabilities, we conducted a calibration analysis on the fine-tuned SciBERT model. Predicted probabilities were compared with observed outcome frequencies across the 3 target classes using calibration plots and quantitative metrics, including expected calibration error and Brier score. Since Longformer achieved similar classification performance, calibration was performed only for SciBERT.

### Ethical Considerations

This study did not involve human participants or the collection of private data. A publicly available dataset of Cochrane PLSs was used, which can be accessed via the Open Science Framework [46].

The use of publicly available data is exempt from ethics review in accordance with the University of Split School of Medicine guidelines and the Croatian Science Foundation project Professionalism in Health – Decision making in practice and research (IP-2019-04-4882) [47]. Therefore, institutional review board approval and informed consent were not required. The dataset used in this study was collected and shared under conditions that permit secondary analysis without additional consent requirements. The data contain no personally identifiable information. All analyzed PLSs are publicly accessible textual documents.

## Results

### Overview

Among the 4405 PLSs from our dataset, 429 (9.7%) had been manually categorized as conclusive, 1203 (27.3%) as inconclusive, and 2773 (63%) as unclear [24]. These PLSs served as input data for our model. To address class imbalance, we applied random undersampling to the training set by selecting an equal number of PLSs from each class (n=429). This ensured that we had a balanced dataset and reduced the risk of biased model learning.

**Figure 1.**
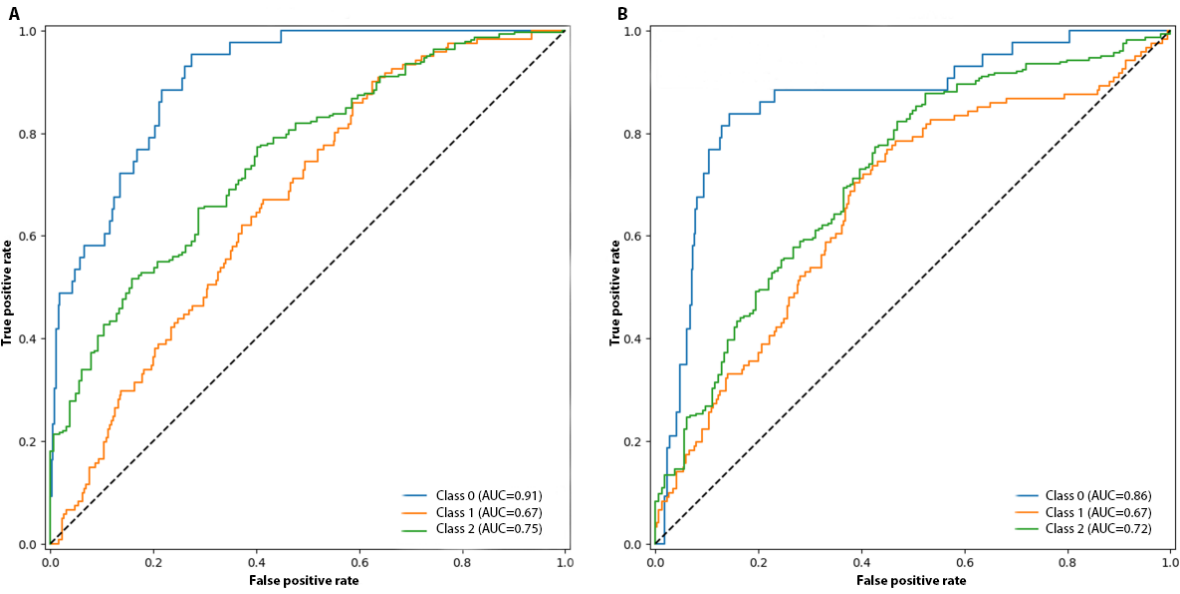
Receiver operating characteristic (ROC) curves and the corresponding area under the curve of the receiver operating characteristic scores for each class (0=conclusive, 1=inconclusive, and 2=unclear); (A) SciBERT model and (B) Longformer model. Calculated and visualized using scikit-learn. AUC: area under the curve.

**Figure 2.**
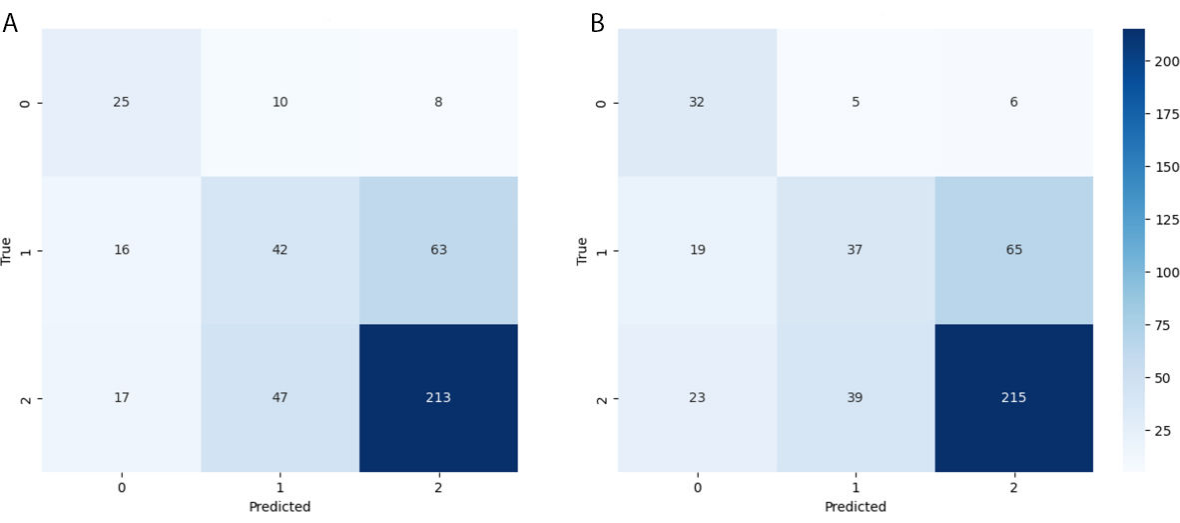
Confusion matrix for the fine-tuned (A) SciBERT and (B) Longformer multiclass classification (0=conclusive, 1=inconclusive, and 2= unclear) used to visualize the performance of the conclusiveness classification algorithm (created using the scikit-learn). The confusion matrix evaluates multiclass performance by comparing the predicted classes with the actual classes. The diagonal elements represent the correct predictions.

In classifying the PLSs, the SciBERT model achieved a balanced accuracy of 56.6%, with AUCROC scores of 0.91 for the conclusive, 0.67 for the inconclusive, and 0.75 for the unclear class. The Longformer model, meanwhile, demonstrated a balanced accuracy of 60.9%, with AUCROC scores of 0.86, 0.67, and 0.72 for the same classes, respectively. The receiver operating characteristic curves and confusion matrices are visualized in Figure 1 and Figure 2, while Table 1 presents a side-by-side comparison of the performance for both models across all classes.

**Table 1.** Per-class performance of the conclusiveness classification for Scientific Bidirectional Encoder Representations from Transformers (SciBERT) and Longformer models^a^.

Model	Precision	Recall	*F*_1_-score	AUCROC^b^ score
Conclusive				
SciBERT	0.43	0.58	0.50	0.91
Longformer	0.43	0.74	0.55	0.87
Inconclusive				
SciBERT	0.42	0.35	0.38	0.67
Longformer	0.46	0.31	0.37	0.67
Unclear				
SciBERT	0.75	0.77	0.76	0.75
Longformer	0.75	0.78	0.76	0.72

a Precision is the proportion of true positives relative to the total of true positives and false positives. Recall is the proportion of true positives relative to the total of true positives and false negatives. *F*_1_-score is the harmonic mean of the precision and recall. Area under the curve of the receiver operating characteristic is the proportion of area below the receiver operating characteristic curve, which is the plot of the true positive rate against the false positive rate. The scores were obtained using the scikit-learn functions.

b AUCROC: area under the curve of the receiver operating characteristic.

For comparison, the baseline SciBERT model achieved a balanced accuracy of only 42.2%, with AUCROC scores of 0.68 for the conclusive, 0.53 for the inconclusive, and 0.53 for the unclear class. The baseline Longformer’s balanced accuracy was 39.0%, with AUCROC scores of 0.69, 0.56, and 0.54 for the same classes, respectively. However, the baseline Longformer was unstable across repeated runs and sometimes predicted only one class. This indicates that the frozen encoder was not able to provide features that separate the 3 categories well. The performance of all models is documented in Multimedia Appendix 1.

### Effect of the Training Set Size and Validation Split on Model Performance

The highest performance was observed with a 10% validation split, whereas 20% and 30% validation splits resulted in similarly reduced accuracies (Table 2).

**Table 2.** Performance of Scientific Bidirectional Encoder Representations from Transformers with varying training and validation splits.

Validation split	Training samples (per class), n	Balanced accuracy (%)
10%	343	56.6
20%	257	53.1
30%	172	53.3

### Manual Validation and Comparison With ChatGPT Performance

Among the 213 PLSs from our additional verification dataset, 44 (20.7%) had been manually categorized as conclusive, 110 (51.6%) as inconclusive, and 59 (27.7%) as unclear. The Cohen κ value between the experts was 0.57, indicating moderate agreement. The baseline GPT-4o model outperformed the trained BERT-based models (Table 3). SciBERT had the poorest performance overall, while ChatGPT had the strongest, with most of its conclusive predictions being correct. ChatGPT also achieved moderate agreement with the human classifications (Cohen κ=0.57). This indicates that ChatGPT’s classifications were as consistent with the expert consensus as an individual expert’s were with one another. In contrast, SciBERT and Longformer demonstrated poor alignment with the reference classifications, with a Cohen κ value of 0.03 and 0.19, respectively, suggesting worse-than-random agreement (Table 3).

**Table 3.** Comparative performance of fine-tuned Scientific Bidirectional Encoder Representations from Transformers (SciBERT) and Longformer models and baseline GPT-4o model on conclusiveness classification task.

Metric	Fine-tuned SciBERT	Fine-tuned Longformer	Baseline GPT-4o
Precision	0.34	0.57	0.74
Recall	0.34	0.44	0.74
*F*_1_-score	0.27	0.40	0.74
Accuracy (%)	34.3	44.1	74.2
Cohen κ^a^	0.03	0.19	0.57

a Predictions were compared against expert annotations, where each plain language summary was manually classified by 2 experts, with a third expert resolving any discrepancies.

### Calibration Analysis

The calibration analysis was performed on the fine-tuned SciBERT model, which was overconfident for the “conclusive” class, poorly calibrated for the “inconclusive” class, and initially underconfident for the “unclear” class, although highly accurate when assigning high probabilities. Calibration plots and quantitative metrics, including expected calibration error and Brier scores (a measure of the average squared difference between predicted probability and actual outcome), are presented in Multimedia Appendix 1.

## Discussion

### Principal Results

Our results showed that transformer-based language models such as SciBERT and Longformer achieved modest performance in classifying Cochrane PLSs based on their level of conclusiveness. Both models were fine-tuned on a balanced dataset and evaluated using standard classification metrics, with Longformer achieving a balanced accuracy of 60.9%, compared with 56.6% for SciBERT. Both models performed best on the conclusive class, achieving relatively high AUCROC and *F*_1_-scores. For the unclear class, SciBERT demonstrated stronger precision and recall. However, both models struggled to distinguish the inconclusive class, with the lowest *F*_1_-scores and overlapping errors with the unclear category. Both models underperformed on the inconclusive class, with poor AUCROC and low precision and recall scores. These findings may indicate that conclusiveness is expressed in linguistically nuanced ways that the models are unable to detect and that there could be a semantic overlap between inconclusive and unclear PLSs. Both models were outperformed by ChatGPT, which achieved better accuracy and interrater agreement. This suggests that general-purpose LLMs may offer more reliable performance for this classification task, even without domain-specific fine-tuning. Notably, GPT-4o achieved the same Cohen κ value (0.57) as the agreement between human experts, suggesting that it mirrors expert-level judgment and nuanced reasoning. This finding highlights the potential of general-purpose LLMs to approximate human evaluation in semantically complex classification tasks.

### Qualitative Insights From Manual Classification

During manual labeling of PLSs, we identified several challenges that may explain why the experts, the fine-tuned models, and ChatGPT all struggled with the classifications. First, there was some ambiguity between the “inconclusive” and “unclear” classes. For example, some PLSs did not clearly state whether the evidence was insufficient, which might be why both human assessors and models were uncertain when assigning these labels. Furthermore, the interpretation of the criteria for the “conclusive” class was occasionally ambiguous, particularly in cases where PLSs included recommendations but lacked clear statements about intervention effectiveness. This ambiguity likely made it difficult for human annotators to determine whether the conclusion was strong enough to classify a PLS as conclusive or inconclusive. Consequently, models trained on these labels may have inherited this ambiguity. We also observed that PLSs included expressions such as “may help” or “probably works,” which are common in scientific writing but can signal uncertainty. This nuance might have been difficult for models to detect, explaining their lower performance in differentiating between inconclusive and unclear statements. These findings suggest that better model instruction, such as through advanced prompt engineering, might help improve future performance of the GPT-4o model. They also highlight the need to incorporate linguistic features of uncertainty more explicitly into the training process.

### Comparison With Prior Work

To our knowledge, there have been no studies on the automatic classification of Cochrane PLSs or full reviews based on the level of conclusiveness, although some studies examined machine learning techniques for making systematic review processes more efficient. For example, one study developed a randomized controlled trial classifier for Cochrane Reviews, a tool that discerns whether a selected study qualifies as a randomized controlled trial [48]. In another, ChatGPT showed strong performance when used for abstractive summarization of longer texts, including news articles and public speeches [49]. However, given the lack of specialized expertise in the field of medicine, ChatGPT does not always grasp the nuances of its terminology and sometimes struggles to recognize important information [50]. In addition, one study found that LLMs sometimes generate factually inconsistent summaries, which could potentially harm readers [51]. Yet, these challenges and the related legal and ethical issues should not discourage the use of LLMs but rather encourage further research and refinement of the technology.

The fine-tuned BERT-based models did not perform well in our classification task, indicating limitations in generalizing to nuanced language in PLSs. In contrast, general-purpose language models can perform better than fine-tuned models in some classification tasks, achieving Cohen κ scores comparable to those of human experts. This is likely because they have been trained on much larger and more diverse text corpora and have more complex architectures, allowing them to better understand context and differentiate linguistic nuances [52]. This is also in line with findings by Davidson and Chae [53] that LLMs, particularly when fine-tuned on prompts that include explicit instructions, can outperform traditional supervised models in a variety of classification settings without task-specific training.

### Limitations

This study has several limitations. First, although we began with a relatively large dataset of 4405 PLSs, the “conclusive” class comprised only about 9.73% (n=429) of the total dataset. To address this issue, we applied random undersampling, which reduced the number of PLSs in the “inconclusive” and “unclear” classes. While this approach ensured balanced class representation, it also removed a substantial amount of data (2344/2773, 84.53% of the “unclear” class) that could have supported more robust model learning. This likely limited the models’ ability to learn the linguistic variability of the majority class. In contrast, GPT-4o was used in zero-shot inference settings and was not trained on our dataset, meaning that its performance could not be affected by the undersampling procedure that constrained the fine-tuned models. Alternative approaches, such as applying class-weighted loss functions or oversampling minority classes, may yield improved performance in future work.

Second, the dataset was based exclusively on PLSs of Cochrane Reviews, representing a single domain within evidence-based health literature. This may also limit the model’s generalizability to other types of health communication.

Third, there were often very subtle linguistic differences between the inconclusive and unclear PLSs, which introduced noise in model classification. Some PLSs lacked clear phrasing or used expressions such as “may help,” and “probably works” that were difficult to interpret consistently, even for human annotators. This ambiguity likely contributed to the models’ difficulty in separating these two classes.

Fourth, although one researcher (IB) participated in and provided instruction for both the original 2019 annotation and the current one, the full annotator teams differed between the two studies. It is possible that there were subtle differences in how annotators interpreted or applied the criteria, raising the possibility of annotator drift between the original labels used for model training and the new labels used for verification. Such drift may partly account for the observed decline in performance of the fine-tuned models on the newer dataset.

Fifth, when comparing accuracy across different splits, it is important to note that altering the training and validation proportion also changes the size of the remaining test set. Because of this, the test benchmarks were not identical across these experiments. While this does not affect the qualitative pattern we observed, the varying test baseline may contribute to numerical differences in accuracy.

Additionally, the fine-tuned SciBERT model showed high volatility in validation loss across training epochs, including abrupt spikes prior to early stopping. This suggests that the model may not have reached a fully stable convergence point, possibly due to the limited size of the training set and the complexity of the task. Such fluctuations may have constrained the model’s performance.

Finally, although we compared our fine-tuned models with GPT-4o, we did not use advanced prompt engineering or task-specific tuning. This likely underestimated ChatGPT’s performance on this classification task.

### Future Work and Recommendations

Future studies should expand the dataset to include PLSs from non-Cochrane sources and from different health domains, which could improve model generalizability. First, although there was no meaningful difference in the performance of different training and validation splits, future work should also explore the impact of larger datasets on model performance. Second, adding task-specific fine-tuning and advanced prompt engineering of LLMs such as GPT-4o could improve classification accuracy even more. Third, models may be able to more successfully differentiate between inconclusive and unclear classes by incorporating linguistic features that capture uncertainty and conclusiveness cues.

In the long term, implementing a general-purpose LLM, such as ChatGPT, within Cochrane platforms (eg, the RevMan Web dashboard) could assist authors in ensuring that their conclusions are clear and guide readers through reviews according to their conclusiveness level. Additionally, LLMs could support users of the Cochrane Library by offering filters or tags that group PLSs by conclusiveness. However, additional model validation and ethical review should precede these real-world applications.

### Conclusions

We explored the use of 2 fine-tuned transformer-based models—SciBERT and Longformer—for classifying Cochrane PLS according to their level of conclusiveness. Both models demonstrated modest internal performance but poor generalization to newly published PLSs, particularly in distinguishing between inconclusive and unclear categories, likely due to their semantic overlap. An empirical analysis of different training and validation splits confirmed that larger training sets improve model performance, although the gains were modest. Most importantly, both models were outperformed by ChatGPT, which as a general-purpose language model, achieved higher accuracy (74%) and agreement with expert annotations, suggesting that state-of-the-art LLMs hold greater potential for health care information dissemination.

## Supplementary material

10.2196/72657 Multimedia Appendix 1 Supplementary material presenting detailed model training procedures, experimental setups, and full performance metrics for SciBERT and Longformer models.

## Abbreviations

AI: artificial intelligence
AUCROC: area under the curve of the receiver operating characteristic
BERT: Bidirectional Encoder Representations from Transformers
LLM: large language model
NLP: natural language processing
PLS: plain language summary
SciBERT: Scientific Bidirectional Encoder Representations from Transformers
