# Supplementary material for: Classification of Cochrane Plain Language Summaries by Conclusiveness Using Transformer-Based Models and ChatGPT: Retrospective Observational Study
Source: JMIR Med Inform. 2026 Apr 14;14:e72657. doi: 10.2196/72657 (PMC13078607; doi:10.2196/72657)
Supplement: Multimedia Appendix 1 [file medinform-v14-e72657-s001.docx]

# 1. SciBERT finetuning

Using device: cuda

Training class distribution (balanced):

Conclusiveness

0 343

1 343

2 343

Name: count, dtype: int64

Training Epoch 1/15: 100%|█████████████████████████████████████████████████████████████| 17/17 [05:11<00:00, 18.34s/it]

Validation: 100%|████████████████████████████████████████████████████████████████████████| 7/7 [00:57<00:00, 8.28s/it]

Epoch 1/15

Training Loss: 1.1669, Accuracy: 0.3547

Validation Loss: 1.0882, Accuracy: 0.3614

Validation loss improved — model saved.

Training Epoch 2/15: 100%|█████████████████████████████████████████████████████████████| 17/17 [04:49<00:00, 17.01s/it]

Validation: 100%|████████████████████████████████████████████████████████████████████████| 7/7 [00:56<00:00, 8.07s/it]

Epoch 2/15

Training Loss: 1.1111, Accuracy: 0.4198

Validation Loss: 1.1007, Accuracy: 0.3477

No improvement. EarlyStopping counter: 1/2

Training Epoch 3/15: 100%|█████████████████████████████████████████████████████████████| 17/17 [04:36<00:00, 16.28s/it]

Validation: 100%|████████████████████████████████████████████████████████████████████████| 7/7 [01:08<00:00, 9.72s/it]

Epoch 3/15

Training Loss: 1.0113, Accuracy: 0.5005

Validation Loss: 0.9380, Accuracy: 0.5523

Validation loss improved — model saved.

Training Epoch 4/15: 82%|██████████████████████████████████████████████████▏ | 14/17 [04:32<00:58, 19.48s/it]Error during conversion: ChunkedEncodingError(ProtocolError('Response ended prematurely'))

Training Epoch 4/15: 100%|█████████████████████████████████████████████████████████████| 17/17 [05:11<00:00, 18.35s/it]

Validation: 100%|████████████████████████████████████████████████████████████████████████| 7/7 [01:01<00:00, 8.84s/it]

Epoch 4/15

Training Loss: 0.8727, Accuracy: 0.5948

Validation Loss: 0.9165, Accuracy: 0.5614

Validation loss improved — model saved.

Training Epoch 5/15: 100%|█████████████████████████████████████████████████████████████| 17/17 [04:51<00:00, 17.16s/it]

Validation: 100%|████████████████████████████████████████████████████████████████████████| 7/7 [00:59<00:00, 8.51s/it]

Epoch 5/15

Training Loss: 0.7842, Accuracy: 0.6511

Validation Loss: 0.7908, Accuracy: 0.6273

Validation loss improved — model saved.

Training Epoch 6/15: 100%|█████████████████████████████████████████████████████████████| 17/17 [04:59<00:00, 17.61s/it]

Validation: 100%|████████████████████████████████████████████████████████████████████████| 7/7 [01:04<00:00, 9.27s/it]

Epoch 6/15

Training Loss: 0.7438, Accuracy: 0.6715

Validation Loss: 1.0607, Accuracy: 0.4795

No improvement. EarlyStopping counter: 1/2

Training Epoch 7/15: 100%|█████████████████████████████████████████████████████████████| 17/17 [04:46<00:00, 16.82s/it]

Validation: 100%|████████████████████████████████████████████████████████████████████████| 7/7 [01:00<00:00, 8.64s/it]

Epoch 7/15

Training Loss: 0.6528, Accuracy: 0.7337

Validation Loss: 1.0350, Accuracy: 0.5591

No improvement. EarlyStopping counter: 2/2

Early stopping triggered.

Best model loaded.


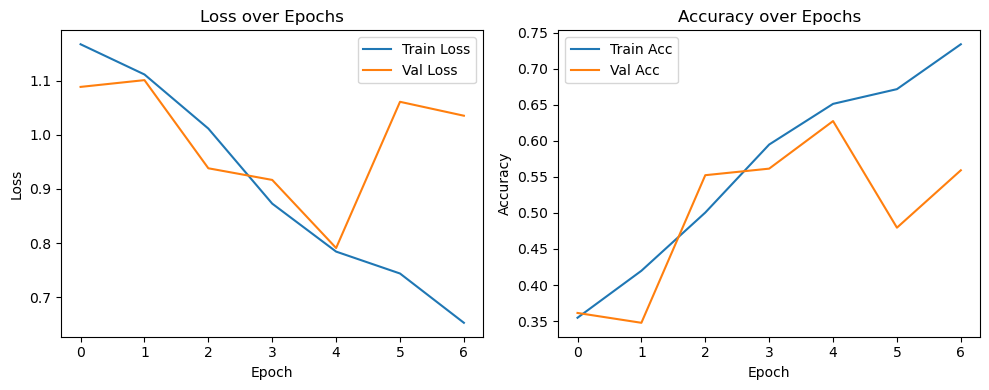


Loaded best model from best_model.pt

Testing: 100%|███████████████████████████████████████████████████████████████████████████| 7/7 [01:30<00:00, 12.96s/it]

Test Accuracy: 0.6349


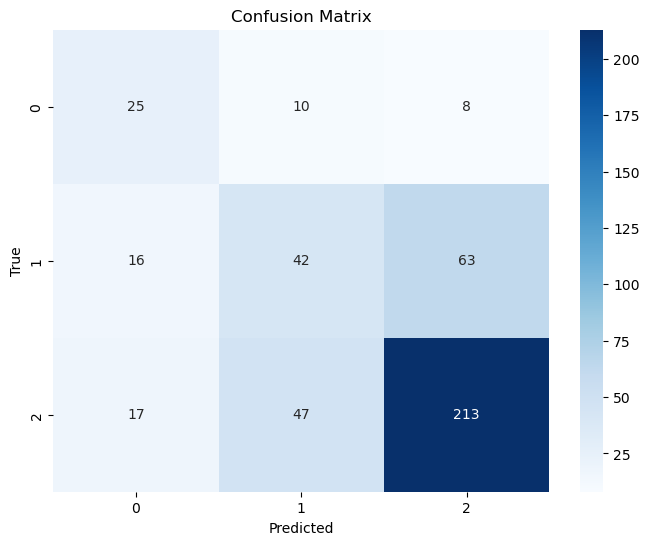


Classification Report:

precision recall f1-score support

0 0.43 0.58 0.50 43

1 0.42 0.35 0.38 121

2 0.75 0.77 0.76 277

accuracy 0.63 441

macro avg 0.54 0.57 0.55 441

weighted avg 0.63 0.63 0.63 441

AUROC for Class 0: 0.906

AUROC for Class 1: 0.667

AUROC for Class 2: 0.751


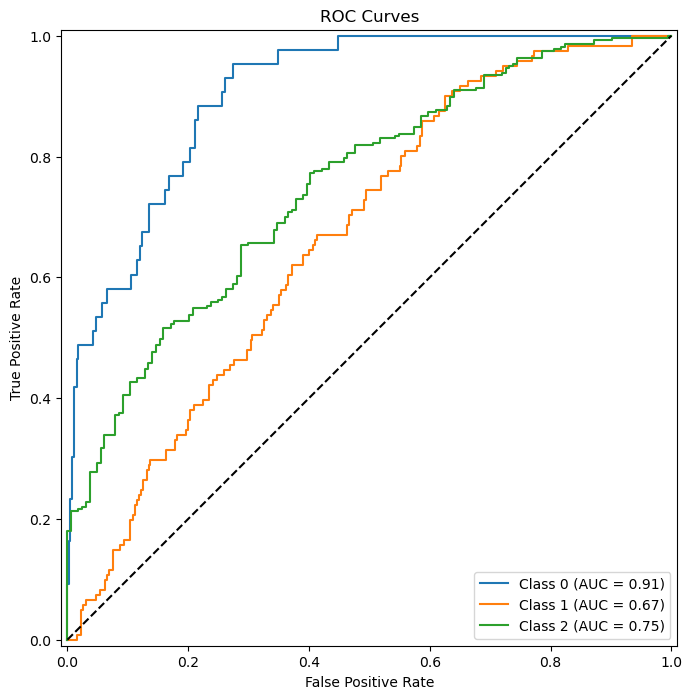


F1 Score (weighted): 0.630

Balanced Accuracy: 0.566

# 2. Longformer finetuning

Using device: cuda

Training class distribution (balanced):

Conclusiveness

0 343

1 343

2 343

Name: count, dtype: int64

Training Epoch 1/10: 100%|███████████████████████████████████████████████████████████| 129/129 [05:05<00:00, 2.37s/it]

Validation: 100%|██████████████████████████████████████████████████████████████████████| 55/55 [00:21<00:00, 2.59it/s]

Epoch 1/10

Training Loss: 1.1494, Accuracy: 0.3431

Validation Loss: 1.2853, Accuracy: 0.1727

Validation loss improved — model saved.

Training Epoch 2/10: 100%|███████████████████████████████████████████████████████████| 129/129 [05:05<00:00, 2.37s/it]

Validation: 100%|██████████████████████████████████████████████████████████████████████| 55/55 [00:21<00:00, 2.59it/s]

Epoch 2/10

Training Loss: 1.0999, Accuracy: 0.3878

Validation Loss: 1.1619, Accuracy: 0.2091

Validation loss improved — model saved.

Training Epoch 3/10: 100%|███████████████████████████████████████████████████████████| 129/129 [05:05<00:00, 2.37s/it]

Validation: 100%|██████████████████████████████████████████████████████████████████████| 55/55 [00:21<00:00, 2.59it/s]

Epoch 3/10

Training Loss: 1.0814, Accuracy: 0.3994

Validation Loss: 1.0869, Accuracy: 0.2727

Validation loss improved — model saved.

Training Epoch 4/10: 100%|███████████████████████████████████████████████████████████| 129/129 [05:08<00:00, 2.40s/it]

Validation: 100%|██████████████████████████████████████████████████████████████████████| 55/55 [00:21<00:00, 2.56it/s]

Epoch 4/10

Training Loss: 1.0009, Accuracy: 0.4966

Validation Loss: 1.0067, Accuracy: 0.5432

Validation loss improved — model saved.

Training Epoch 5/10: 100%|███████████████████████████████████████████████████████████| 129/129 [05:05<00:00, 2.37s/it]

Validation: 100%|██████████████████████████████████████████████████████████████████████| 55/55 [00:21<00:00, 2.58it/s]

Epoch 5/10

Training Loss: 0.8974, Accuracy: 0.5462

Validation Loss: 1.0712, Accuracy: 0.5545

No improvement. EarlyStopping counter: 1/3

Training Epoch 6/10: 100%|███████████████████████████████████████████████████████████| 129/129 [05:05<00:00, 2.37s/it]

Validation: 100%|██████████████████████████████████████████████████████████████████████| 55/55 [00:21<00:00, 2.59it/s]

Epoch 6/10

Training Loss: 0.8142, Accuracy: 0.6152

Validation Loss: 0.9249, Accuracy: 0.6295

Validation loss improved — model saved.

Training Epoch 7/10: 100%|███████████████████████████████████████████████████████████| 129/129 [05:05<00:00, 2.37s/it]

Validation: 100%|██████████████████████████████████████████████████████████████████████| 55/55 [00:21<00:00, 2.59it/s]

Epoch 7/10

Training Loss: 0.7157, Accuracy: 0.7007

Validation Loss: 0.9865, Accuracy: 0.6409

No improvement. EarlyStopping counter: 1/3

Training Epoch 8/10: 100%|███████████████████████████████████████████████████████████| 129/129 [05:05<00:00, 2.37s/it]

Validation: 100%|██████████████████████████████████████████████████████████████████████| 55/55 [00:21<00:00, 2.59it/s]

Epoch 8/10

Training Loss: 0.6274, Accuracy: 0.7716

Validation Loss: 0.9292, Accuracy: 0.6636

No improvement. EarlyStopping counter: 2/3

Training Epoch 9/10: 100%|███████████████████████████████████████████████████████████| 129/129 [05:06<00:00, 2.38s/it]

Validation: 100%|██████████████████████████████████████████████████████████████████████| 55/55 [00:21<00:00, 2.59it/s]

Epoch 9/10

Training Loss: 0.5452, Accuracy: 0.8270

Validation Loss: 1.0147, Accuracy: 0.6227

No improvement. EarlyStopping counter: 3/3

Early stopping triggered.

Best model loaded.


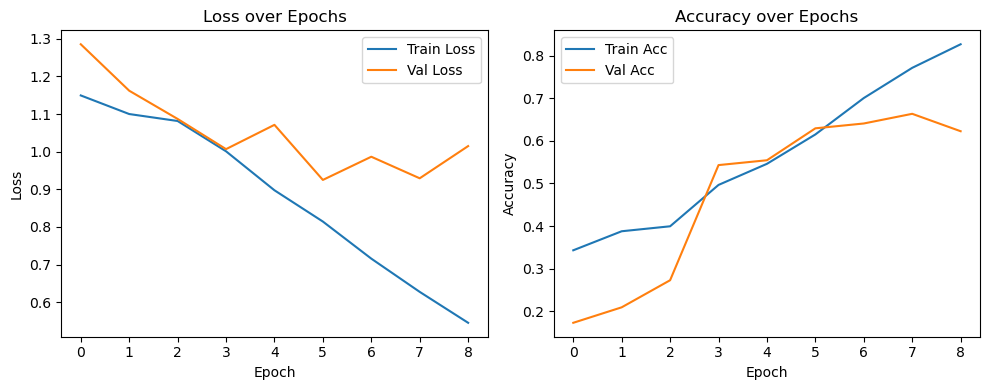


Loaded best model from best_model.pt

Testing: 100%|█████████████████████████████████████████████████████████████████████████| 56/56 [00:21<00:00, 2.62it/s]

Test Accuracy: 0.6440


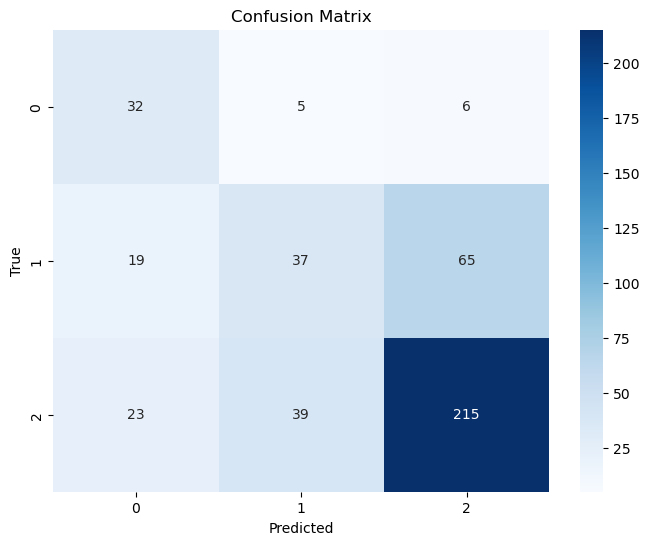


Classification Report:

precision recall f1-score support

0 0.43 0.74 0.55 43

1 0.46 0.31 0.37 121

2 0.75 0.78 0.76 277

accuracy 0.64 441

macro avg 0.55 0.61 0.56 441

weighted avg 0.64 0.64 0.63 441

AUROC for Class 0: 0.862

AUROC for Class 1: 0.666

AUROC for Class 2: 0.720


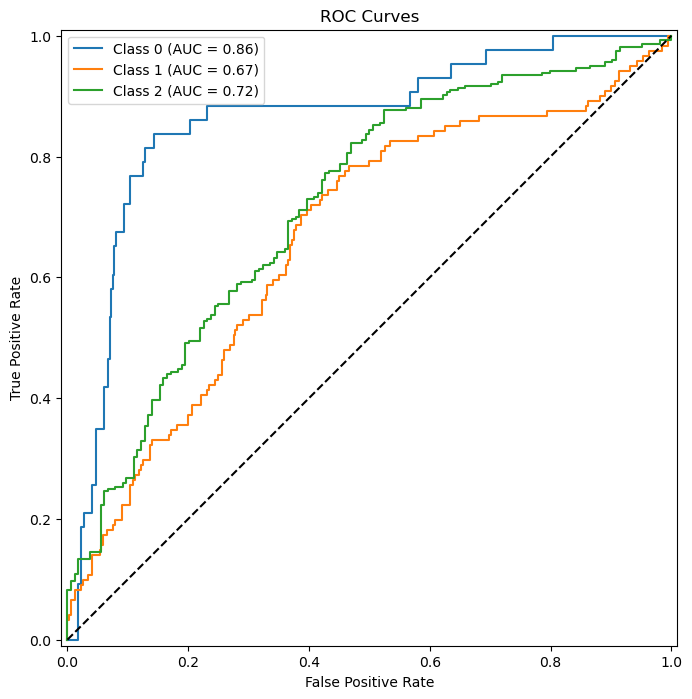


F1 Score (weighted): 0.634

Balanced Accuracy: 0.609

# 3. Testing finetuned SciBERT model with different training/validation split

## 3.1. 60% training, 20% testing, and 20% validation split

Using device: cuda

Training class distribution (balanced):

Conclusiveness

2 257

1 257

0 257

Name: count, dtype: int64

Training Epoch 1/15: 100%|█████████████████████████████████████████████████████████████| 13/13 [05:47<00:00, 26.72s/it]

Validation: 100%|██████████████████████████████████████████████████████████████████████| 14/14 [01:34<00:00, 6.75s/it]

Epoch 1/15

Training Loss: 1.1413, Accuracy: 0.3502

Validation Loss: 1.1542, Accuracy: 0.2304

Validation loss improved — model saved.

Training Epoch 2/15: 100%|█████████████████████████████████████████████████████████████| 13/13 [05:12<00:00, 24.03s/it]

Validation: 100%|██████████████████████████████████████████████████████████████████████| 14/14 [01:42<00:00, 7.33s/it]

Epoch 2/15

Training Loss: 1.0967, Accuracy: 0.3696

Validation Loss: 1.1247, Accuracy: 0.2951

Validation loss improved — model saved.

Training Epoch 3/15: 100%|█████████████████████████████████████████████████████████████| 13/13 [05:07<00:00, 23.64s/it]

Validation: 100%|██████████████████████████████████████████████████████████████████████| 14/14 [01:42<00:00, 7.30s/it]

Epoch 3/15

Training Loss: 1.0460, Accuracy: 0.4578

Validation Loss: 1.0051, Accuracy: 0.4688

Validation loss improved — model saved.

Training Epoch 4/15: 100%|█████████████████████████████████████████████████████████████| 13/13 [04:57<00:00, 22.92s/it]

Validation: 100%|██████████████████████████████████████████████████████████████████████| 14/14 [01:35<00:00, 6.80s/it]

Epoch 4/15

Training Loss: 0.9433, Accuracy: 0.5357

Validation Loss: 0.9563, Accuracy: 0.5426

Validation loss improved — model saved.

Training Epoch 5/15: 100%|█████████████████████████████████████████████████████████████| 13/13 [05:03<00:00, 23.35s/it]

Validation: 100%|██████████████████████████████████████████████████████████████████████| 14/14 [01:39<00:00, 7.08s/it]

Epoch 5/15

Training Loss: 0.8150, Accuracy: 0.5953

Validation Loss: 1.0953, Accuracy: 0.4688

No improvement. EarlyStopping counter: 1/2

Training Epoch 6/15: 100%|█████████████████████████████████████████████████████████████| 13/13 [04:49<00:00, 22.31s/it]

Validation: 100%|██████████████████████████████████████████████████████████████████████| 14/14 [01:36<00:00, 6.90s/it]

Epoch 6/15

Training Loss: 0.7349, Accuracy: 0.6654

Validation Loss: 1.0072, Accuracy: 0.5221

No improvement. EarlyStopping counter: 2/2

Early stopping triggered.

Best model loaded.


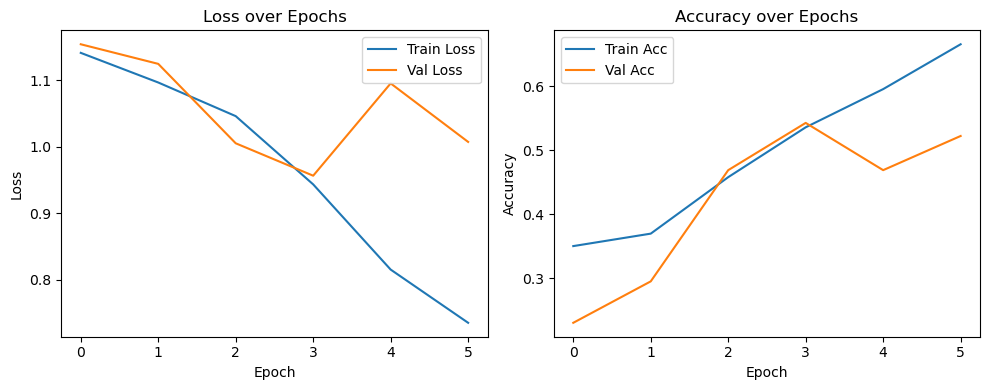


Loaded best model from best_model.pt

Testing: 100%|█████████████████████████████████████████████████████████████████████████| 14/14 [02:47<00:00, 11.95s/it]

Test Accuracy: 0.5562


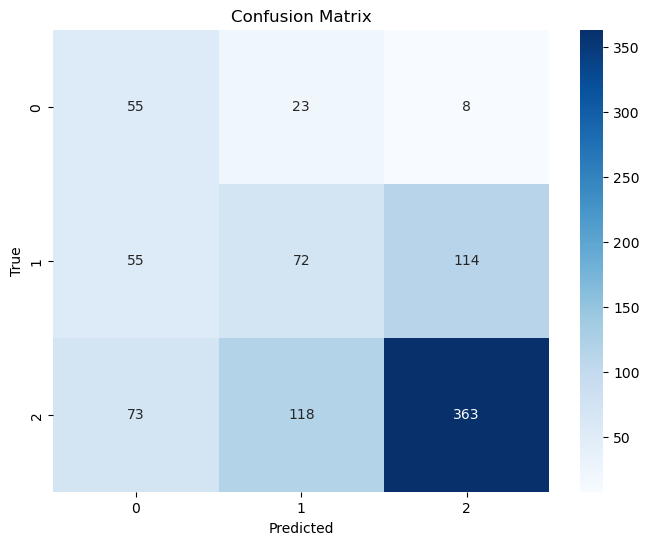


Classification Report:

precision recall f1-score support

0 0.30 0.64 0.41 86

1 0.34 0.30 0.32 241

2 0.75 0.66 0.70 554

accuracy 0.56 881

macro avg 0.46 0.53 0.47 881

weighted avg 0.59 0.56 0.57 881

AUROC for Class 0: 0.826

AUROC for Class 1: 0.591

AUROC for Class 2: 0.698


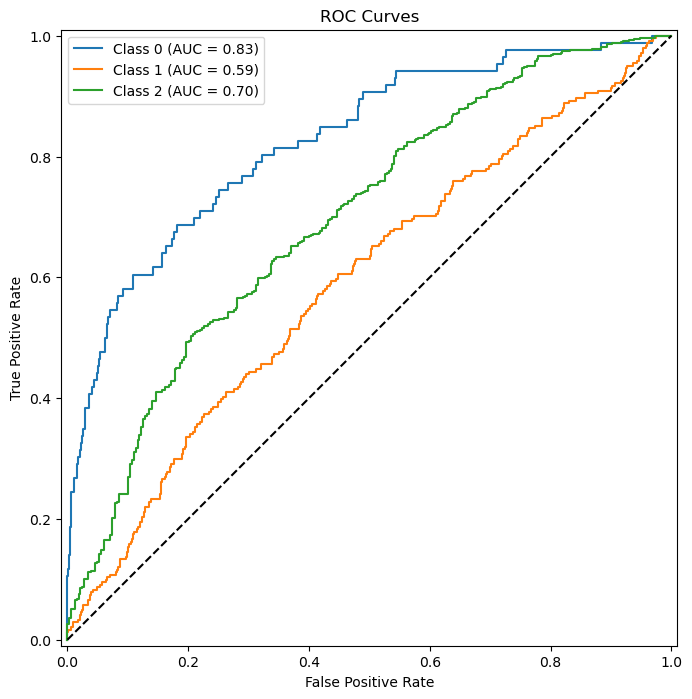


F1 Score (weighted): 0.566

Balanced Accuracy: 0.531

## 3.2. 40% training, 30% testing, and 30% validation split

Using device: cuda

Training class distribution (balanced):

Conclusiveness

1 172

2 172

0 172

Name: count, dtype: int64

Training Epoch 1/15: 100%|███████████████████████████████████████████████████████████████| 9/9 [03:11<00:00, 21.28s/it]

Validation: 100%|██████████████████████████████████████████████████████████████████████| 21/21 [01:52<00:00, 5.35s/it]

Epoch 1/15

Training Loss: 1.1714, Accuracy: 0.3605

Validation Loss: 1.0275, Accuracy: 0.5791

Validation loss improved — model saved.

Training Epoch 2/15: 100%|███████████████████████████████████████████████████████████████| 9/9 [03:11<00:00, 21.29s/it]

Validation: 100%|██████████████████████████████████████████████████████████████████████| 21/21 [01:54<00:00, 5.46s/it]

Epoch 2/15

Training Loss: 1.1057, Accuracy: 0.3876

Validation Loss: 1.1475, Accuracy: 0.2157

No improvement. EarlyStopping counter: 1/2

Training Epoch 3/15: 100%|███████████████████████████████████████████████████████████████| 9/9 [03:12<00:00, 21.37s/it]

Validation: 100%|██████████████████████████████████████████████████████████████████████| 21/21 [01:52<00:00, 5.34s/it]

Epoch 3/15

Training Loss: 1.0327, Accuracy: 0.4302

Validation Loss: 0.9564, Accuracy: 0.5534

Validation loss improved — model saved.

Training Epoch 4/15: 100%|███████████████████████████████████████████████████████████████| 9/9 [03:11<00:00, 21.28s/it]

Validation: 100%|██████████████████████████████████████████████████████████████████████| 21/21 [01:52<00:00, 5.36s/it]

Epoch 4/15

Training Loss: 0.9716, Accuracy: 0.5136

Validation Loss: 1.1537, Accuracy: 0.3293

No improvement. EarlyStopping counter: 1/2

Training Epoch 5/15: 100%|███████████████████████████████████████████████████████████████| 9/9 [03:11<00:00, 21.29s/it]

Validation: 100%|██████████████████████████████████████████████████████████████████████| 21/21 [01:52<00:00, 5.34s/it]

Epoch 5/15

Training Loss: 0.9008, Accuracy: 0.5678

Validation Loss: 0.8457, Accuracy: 0.6291

Validation loss improved — model saved.

Training Epoch 6/15: 100%|███████████████████████████████████████████████████████████████| 9/9 [03:11<00:00, 21.30s/it]

Validation: 100%|██████████████████████████████████████████████████████████████████████| 21/21 [01:50<00:00, 5.26s/it]

Epoch 6/15

Training Loss: 0.8564, Accuracy: 0.5891

Validation Loss: 1.1070, Accuracy: 0.4542

No improvement. EarlyStopping counter: 1/2

Training Epoch 7/15: 100%|███████████████████████████████████████████████████████████████| 9/9 [03:11<00:00, 21.29s/it]

Validation: 100%|██████████████████████████████████████████████████████████████████████| 21/21 [01:51<00:00, 5.33s/it]

Epoch 7/15

Training Loss: 0.7701, Accuracy: 0.6512

Validation Loss: 0.8431, Accuracy: 0.6109

Validation loss improved — model saved.

Training Epoch 8/15: 100%|███████████████████████████████████████████████████████████████| 9/9 [03:11<00:00, 21.29s/it]

Validation: 100%|██████████████████████████████████████████████████████████████████████| 21/21 [01:52<00:00, 5.34s/it]

Epoch 8/15

Training Loss: 0.6888, Accuracy: 0.6957

Validation Loss: 0.9710, Accuracy: 0.5420

No improvement. EarlyStopping counter: 1/2

Training Epoch 9/15: 100%|███████████████████████████████████████████████████████████████| 9/9 [03:11<00:00, 21.27s/it]

Validation: 100%|██████████████████████████████████████████████████████████████████████| 21/21 [01:52<00:00, 5.35s/it]

Epoch 9/15

Training Loss: 0.5477, Accuracy: 0.7733

Validation Loss: 0.8704, Accuracy: 0.5920

No improvement. EarlyStopping counter: 2/2

Early stopping triggered.

Best model loaded.


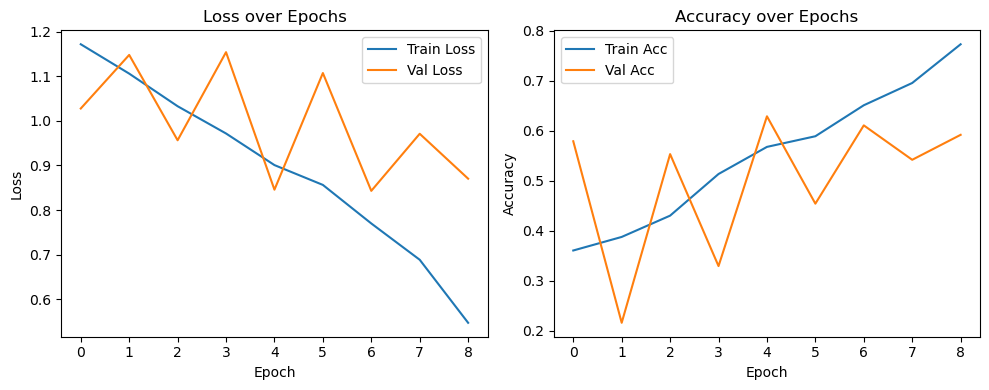


Loaded best model from best_model.pt

Testing: 100%|█████████████████████████████████████████████████████████████████████████| 21/21 [03:10<00:00, 9.05s/it]

Test Accuracy: 0.5809


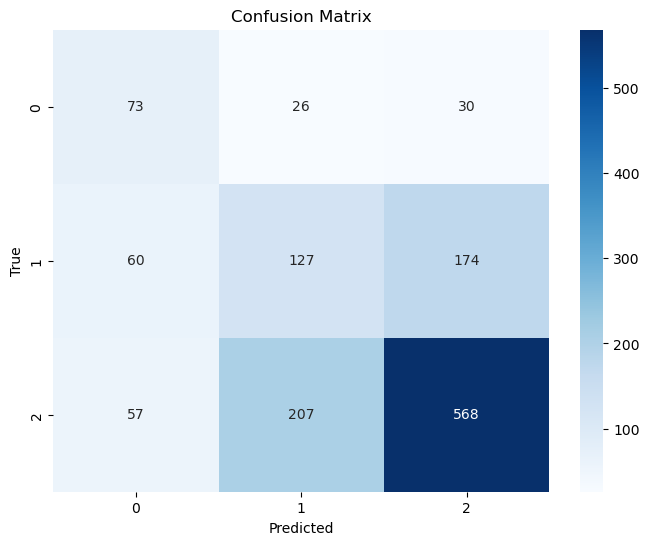


Classification Report:

precision recall f1-score support

0 0.38 0.57 0.46 129

1 0.35 0.35 0.35 361

2 0.74 0.68 0.71 832

accuracy 0.58 1322

macro avg 0.49 0.53 0.51 1322

weighted avg 0.60 0.58 0.59 1322

AUROC for Class 0: 0.832

AUROC for Class 1: 0.587

AUROC for Class 2: 0.703


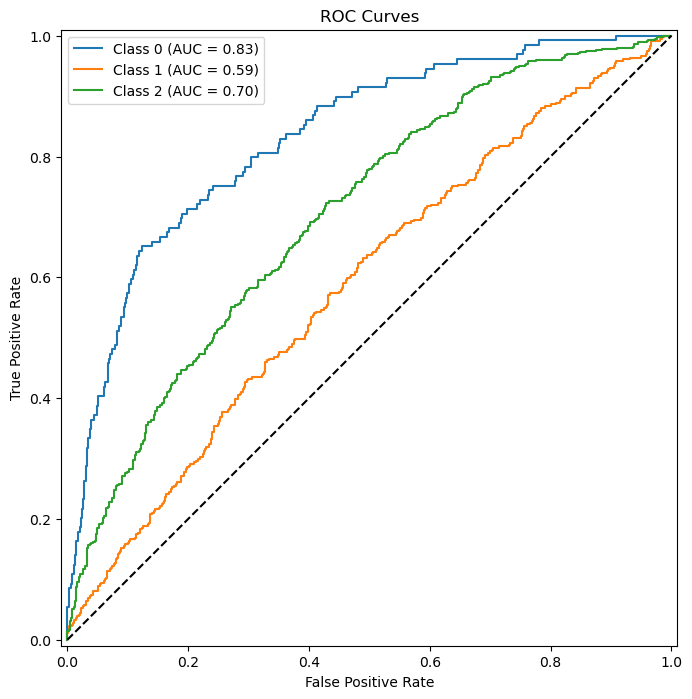


F1 Score (weighted): 0.587

Balanced Accuracy: 0.533

# 4. Baseline SciBERT

Using device: cuda

Training class distribution (balanced):

Conclusiveness

0 343

1 343

2 343

Name: count, dtype: int64

Training Epoch 1/15: 100%|█████████████████████████████████████████████████████████████| 17/17 [00:07<00:00, 2.18it/s]

Validation: 100%|████████████████████████████████████████████████████████████████████████| 7/7 [00:03<00:00, 2.17it/s]

Epoch 1/15

Training Loss: 1.2444, Accuracy: 0.3615

Validation Loss: 1.3615, Accuracy: 0.2773

Validation loss improved — model saved.

Training Epoch 2/15: 100%|█████████████████████████████████████████████████████████████| 17/17 [00:07<00:00, 2.22it/s]

Validation: 100%|████████████████████████████████████████████████████████████████████████| 7/7 [00:03<00:00, 2.21it/s]

Epoch 2/15

Training Loss: 1.1889, Accuracy: 0.3673

Validation Loss: 1.2224, Accuracy: 0.2886

Validation loss improved — model saved.

Training Epoch 3/15: 100%|█████████████████████████████████████████████████████████████| 17/17 [00:07<00:00, 2.21it/s]

Validation: 100%|████████████████████████████████████████████████████████████████████████| 7/7 [00:03<00:00, 2.20it/s]

Epoch 3/15

Training Loss: 1.1568, Accuracy: 0.3596

Validation Loss: 1.1440, Accuracy: 0.2864

Validation loss improved — model saved.

Training Epoch 4/15: 100%|█████████████████████████████████████████████████████████████| 17/17 [00:07<00:00, 2.21it/s]

Validation: 100%|████████████████████████████████████████████████████████████████████████| 7/7 [00:03<00:00, 2.19it/s]

Epoch 4/15

Training Loss: 1.1506, Accuracy: 0.3343

Validation Loss: 1.1108, Accuracy: 0.3182

Validation loss improved — model saved.

Training Epoch 5/15: 100%|█████████████████████████████████████████████████████████████| 17/17 [00:07<00:00, 2.21it/s]

Validation: 100%|████████████████████████████████████████████████████████████████████████| 7/7 [00:03<00:00, 2.19it/s]

Epoch 5/15

Training Loss: 1.1300, Accuracy: 0.3518

Validation Loss: 1.0992, Accuracy: 0.3750

Validation loss improved — model saved.

Training Epoch 6/15: 100%|█████████████████████████████████████████████████████████████| 17/17 [00:07<00:00, 2.20it/s]

Validation: 100%|████████████████████████████████████████████████████████████████████████| 7/7 [00:03<00:00, 2.19it/s]

Epoch 6/15

Training Loss: 1.1388, Accuracy: 0.3469

Validation Loss: 1.0907, Accuracy: 0.3773

Validation loss improved — model saved.

Training Epoch 7/15: 100%|█████████████████████████████████████████████████████████████| 17/17 [00:07<00:00, 2.19it/s]

Validation: 100%|████████████████████████████████████████████████████████████████████████| 7/7 [00:03<00:00, 2.14it/s]

Epoch 7/15

Training Loss: 1.1307, Accuracy: 0.3469

Validation Loss: 1.0906, Accuracy: 0.3727

Validation loss improved — model saved.

Training Epoch 8/15: 100%|█████████████████████████████████████████████████████████████| 17/17 [00:07<00:00, 2.17it/s]

Validation: 100%|████████████████████████████████████████████████████████████████████████| 7/7 [00:03<00:00, 2.16it/s]

Epoch 8/15

Training Loss: 1.1586, Accuracy: 0.3537

Validation Loss: 1.0856, Accuracy: 0.3705

Validation loss improved — model saved.

Training Epoch 9/15: 100%|█████████████████████████████████████████████████████████████| 17/17 [00:07<00:00, 2.18it/s]

Validation: 100%|████████████████████████████████████████████████████████████████████████| 7/7 [00:03<00:00, 2.16it/s]

Epoch 9/15

Training Loss: 1.1614, Accuracy: 0.3401

Validation Loss: 1.0877, Accuracy: 0.3659

No improvement. EarlyStopping counter: 1/2

Training Epoch 10/15: 100%|████████████████████████████████████████████████████████████| 17/17 [00:07<00:00, 2.17it/s]

Validation: 100%|████████████████████████████████████████████████████████████████████████| 7/7 [00:03<00:00, 2.15it/s]

Epoch 10/15

Training Loss: 1.1655, Accuracy: 0.3479

Validation Loss: 1.0849, Accuracy: 0.3795

Validation loss improved — model saved.

Training Epoch 11/15: 100%|████████████████████████████████████████████████████████████| 17/17 [00:07<00:00, 2.15it/s]

Validation: 100%|████████████████████████████████████████████████████████████████████████| 7/7 [00:03<00:00, 2.15it/s]

Epoch 11/15

Training Loss: 1.1545, Accuracy: 0.3567

Validation Loss: 1.0875, Accuracy: 0.3705

No improvement. EarlyStopping counter: 1/2

Training Epoch 12/15: 100%|████████████████████████████████████████████████████████████| 17/17 [00:07<00:00, 2.16it/s]

Validation: 100%|████████████████████████████████████████████████████████████████████████| 7/7 [00:03<00:00, 2.15it/s]

Epoch 12/15

Training Loss: 1.1234, Accuracy: 0.3887

Validation Loss: 1.0841, Accuracy: 0.3727

Validation loss improved — model saved.

Training Epoch 13/15: 100%|████████████████████████████████████████████████████████████| 17/17 [00:07<00:00, 2.17it/s]

Validation: 100%|████████████████████████████████████████████████████████████████████████| 7/7 [00:03<00:00, 2.15it/s]

Epoch 13/15

Training Loss: 1.1612, Accuracy: 0.3353

Validation Loss: 1.0847, Accuracy: 0.3614

No improvement. EarlyStopping counter: 1/2

Training Epoch 14/15: 100%|████████████████████████████████████████████████████████████| 17/17 [00:07<00:00, 2.16it/s]

Validation: 100%|████████████████████████████████████████████████████████████████████████| 7/7 [00:03<00:00, 2.15it/s]

Epoch 14/15

Training Loss: 1.1488, Accuracy: 0.3605

Validation Loss: 1.0842, Accuracy: 0.3614

No improvement. EarlyStopping counter: 2/2

Early stopping triggered.

Best model loaded.


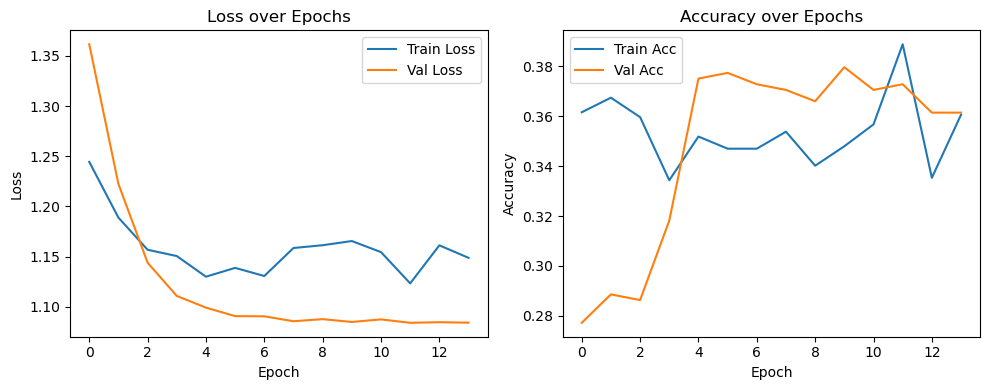


Loaded best model from best_model.pt

Testing: 100%|███████████████████████████████████████████████████████████████████████████| 7/7 [00:03<00:00, 2.15it/s]

Test Accuracy: 0.4036


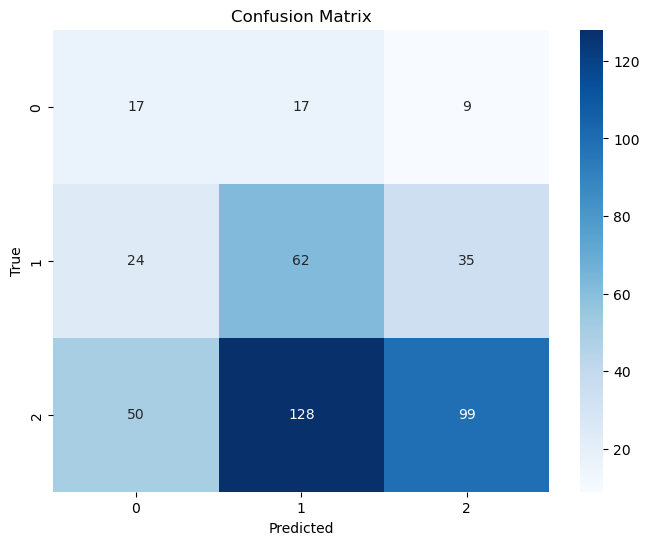


Classification Report:

precision recall f1-score support

0 0.19 0.40 0.25 43

1 0.30 0.51 0.38 121

2 0.69 0.36 0.47 277

accuracy 0.40 441

macro avg 0.39 0.42 0.37 441

weighted avg 0.54 0.40 0.42 441

AUROC for Class 0: 0.675

AUROC for Class 1: 0.526

AUROC for Class 2: 0.526


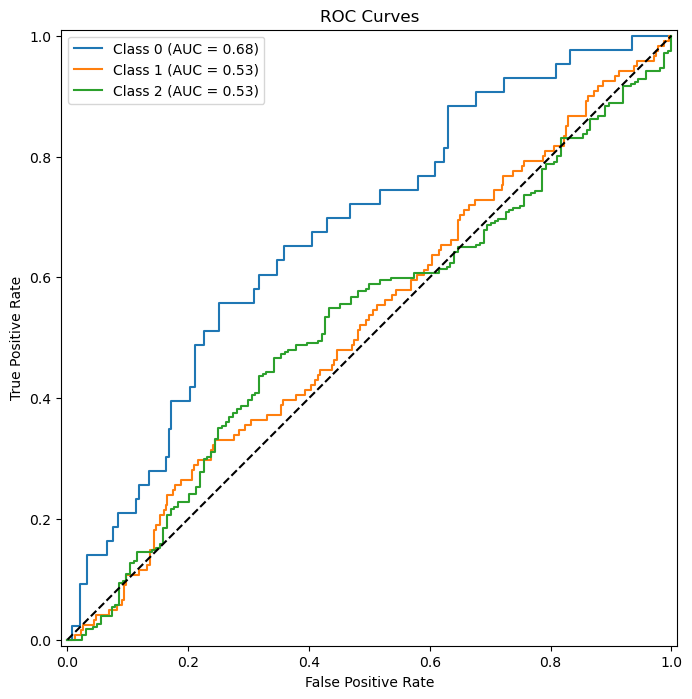


F1 Score (weighted): 0.425

Balanced Accuracy: 0.422

# 5. Baseline Longformer

Using device: cuda

Training class distribution (balanced):

Conclusiveness

0 343

1 343

2 343

Name: count, dtype: int64

Training Epoch 1/10: 100%|███████████████████████████████████████████████████████████| 129/129 [01:19<00:00, 1.62it/s]

Validation: 100%|██████████████████████████████████████████████████████████████████████| 55/55 [00:32<00:00, 1.69it/s]

Epoch 1/10

Training Loss: 1.1795, Accuracy: 0.3392

Validation Loss: 1.3145, Accuracy: 0.0977

Validation loss improved — model saved.

Training Epoch 2/10: 100%|███████████████████████████████████████████████████████████| 129/129 [01:19<00:00, 1.62it/s]

Validation: 100%|██████████████████████████████████████████████████████████████████████| 55/55 [00:32<00:00, 1.68it/s]

Epoch 2/10

Training Loss: 1.1638, Accuracy: 0.3343

Validation Loss: 1.2531, Accuracy: 0.1068

Validation loss improved — model saved.

Training Epoch 3/10: 100%|███████████████████████████████████████████████████████████| 129/129 [01:20<00:00, 1.61it/s]

Validation: 100%|██████████████████████████████████████████████████████████████████████| 55/55 [00:32<00:00, 1.67it/s]

Epoch 3/10

Training Loss: 1.1459, Accuracy: 0.3343

Validation Loss: 1.2119, Accuracy: 0.1182

Validation loss improved — model saved.

Training Epoch 4/10: 100%|███████████████████████████████████████████████████████████| 129/129 [01:20<00:00, 1.61it/s]

Validation: 100%|██████████████████████████████████████████████████████████████████████| 55/55 [00:32<00:00, 1.67it/s]

Epoch 4/10

Training Loss: 1.1404, Accuracy: 0.3333

Validation Loss: 1.1844, Accuracy: 0.1409

Validation loss improved — model saved.

Training Epoch 5/10: 100%|███████████████████████████████████████████████████████████| 129/129 [01:19<00:00, 1.62it/s]

Validation: 100%|██████████████████████████████████████████████████████████████████████| 55/55 [00:32<00:00, 1.68it/s]

Epoch 5/10

Training Loss: 1.1346, Accuracy: 0.3256

Validation Loss: 1.1656, Accuracy: 0.1432

Validation loss improved — model saved.

Training Epoch 6/10: 100%|███████████████████████████████████████████████████████████| 129/129 [01:20<00:00, 1.60it/s]

Validation: 100%|██████████████████████████████████████████████████████████████████████| 55/55 [00:32<00:00, 1.67it/s]

Epoch 6/10

Training Loss: 1.1203, Accuracy: 0.3469

Validation Loss: 1.1518, Accuracy: 0.1614

Validation loss improved — model saved.

Training Epoch 7/10: 100%|███████████████████████████████████████████████████████████| 129/129 [01:20<00:00, 1.61it/s]

Validation: 100%|██████████████████████████████████████████████████████████████████████| 55/55 [00:32<00:00, 1.67it/s]

Epoch 7/10

Training Loss: 1.1278, Accuracy: 0.3275

Validation Loss: 1.1429, Accuracy: 0.1705

Validation loss improved — model saved.

Training Epoch 8/10: 100%|███████████████████████████████████████████████████████████| 129/129 [01:20<00:00, 1.61it/s]

Validation: 100%|██████████████████████████████████████████████████████████████████████| 55/55 [00:32<00:00, 1.67it/s]

Epoch 8/10

Training Loss: 1.1262, Accuracy: 0.3265

Validation Loss: 1.1365, Accuracy: 0.1705

Validation loss improved — model saved.

Training Epoch 9/10: 100%|███████████████████████████████████████████████████████████| 129/129 [01:20<00:00, 1.60it/s]

Validation: 100%|██████████████████████████████████████████████████████████████████████| 55/55 [00:32<00:00, 1.67it/s]

Epoch 9/10

Training Loss: 1.1106, Accuracy: 0.3547

Validation Loss: 1.1333, Accuracy: 0.1841

Validation loss improved — model saved.

Training Epoch 10/10: 100%|██████████████████████████████████████████████████████████| 129/129 [01:20<00:00, 1.60it/s]

Validation: 100%|██████████████████████████████████████████████████████████████████████| 55/55 [00:32<00:00, 1.67it/s]

Epoch 10/10

Training Loss: 1.1173, Accuracy: 0.3324

Validation Loss: 1.1320, Accuracy: 0.1841

Validation loss improved — model saved.

Best model loaded.


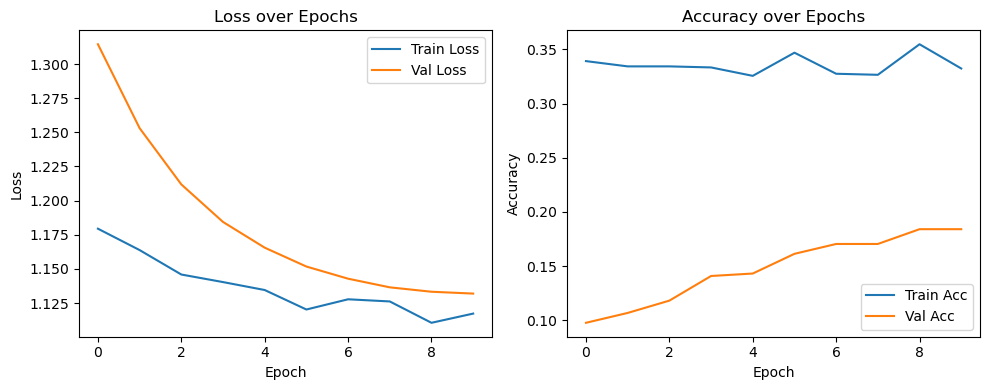


Loaded best model from best_model.pt

Testing: 100%|█████████████████████████████████████████████████████████████████████████| 56/56 [00:33<00:00, 1.70it/s]

Test Accuracy: 0.1769


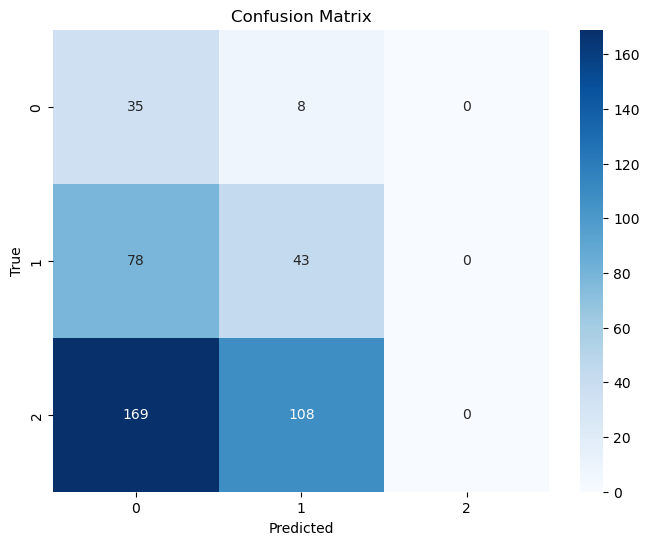


Classification Report:

precision recall f1-score support

0 0.12 0.81 0.22 43

1 0.27 0.36 0.31 121

2 0.00 0.00 0.00 277

accuracy 0.18 441

macro avg 0.13 0.39 0.17 441

weighted avg 0.09 0.18 0.11 441

AUROC for Class 0: 0.691

AUROC for Class 1: 0.556

AUROC for Class 2: 0.543


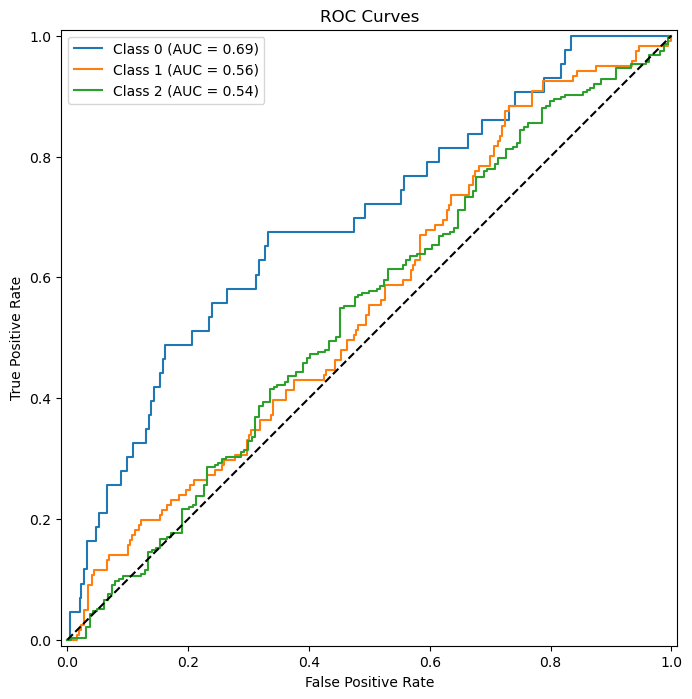


F1 Score (weighted): 0.105

Balanced Accuracy: 0.390

# 6. ChatGPT prompt

I will upload a Cochrane Plain Language Summary (PLS). Classify the PLS based on conclusiveness (conclusive, inconclusive, unclear). Bellow is the explanation of the classes.

Conclusive:

- Positive – signifies the existence of moderate or high-quality evidence supporting the effectiveness/safety.
- Negative – indicates the presence of moderate or high-quality evidence of intervention’s ineffectiveness/harm.
- Equal – denotes that the analysed interventions were of equal effectiveness/safety.

Inconclusive:

- Positive inconclusive – implies the existence of evidence supporting effectiveness/safety, yet the evidence is of low quality/inconclusive and the authors suggest that more research is needed.
- Negative inconclusive – suggests there is evidence of ineffectiveness/harm (indicating that the observed effect or the intervention was unsafe), yet the evidence is low quality/inconclusive. Authors may advise against the intervention/comparison and state that more research is required.
- Equal inconclusive – indicates that the interventions exhibit comparable levels of effectiveness/safety; yet evidence is of a low quality/inconclusive, and the authors emphasize that more research is required.

Unclear:

- No opinion – the authors provided no opinion.
- No evidence – there is no evidence from RCTs because the literature search did not result in any eligible studies, i.e. empty reviews.
- Unclear – authors did not present clear conclusions.

# 7. Calibration Statistics – SciBERT


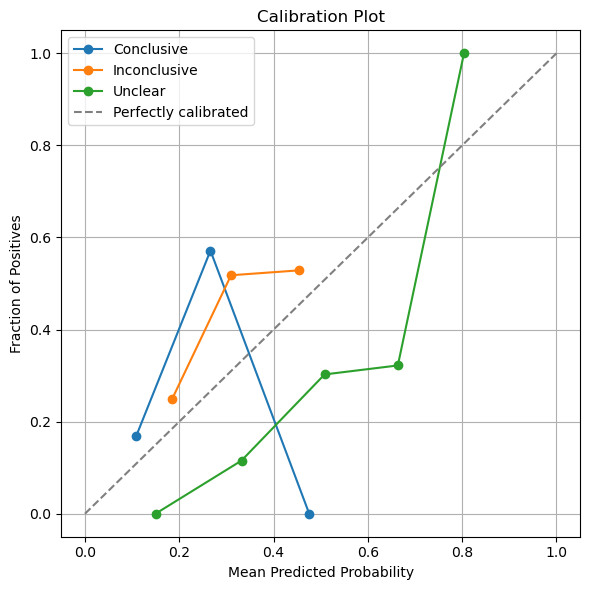


Brier Scores:

Conclusive: 0.1599

Inconclusive: 0.2811

Unclear: 0.2539

Expected Calibration Error (ECE):

Conclusive: 0.0880

Inconclusive: 0.1617

Unclear: 0.2425
